# Dr Zelra Malan (13 November 1963 – 22 November 2025)

**DOI:** 10.4102/phcfm.v18i1.5408

**Published:** 2026-03-13

**Authors:** Felicia Christians

**Affiliations:** 1 Department of Medical Sciences, Division of Family and Community Medicine, Faculty of Health Sciences and Veterinary Medicine, University of Namibia, Windhoek, Namibia

With the passing of Dr Zelra Malan on 22 November 2025, the family medicine community in Africa lost a respected teacher, accomplished scholar and mentor.

Dr Malan qualified as a medical doctor in 1987 and established a career in private practice, where she built trusting relationships with patients. She later completed a Master of Medicine (MMed) in Family Medicine at the University of Pretoria in 2008, marking the start of her academic career. She joined the Division of Family Medicine and Primary Care at Stellenbosch University and completed her Doctor of Philosophy (PhD) in 2015 under Professor Bob Mash. Her doctoral research focused on the development, implementation and evaluation of brief behaviour change counselling (BBCC) for primary care providers, informed by motivational interviewing.

At Stellenbosch University, she developed BBCC into an accredited short course and trained primary care providers across several African countries. She joined the international network of motivational interviewing trainers and led work on embedding behaviour change counselling in routine primary care. From 2014 to 2017, she was co-investigator and project coordinator for a European Union–funded initiative to strengthen primary health care through family physicians in South Africa. Her contributions included leading the development of a national curriculum for a Postgraduate Diploma in Family Medicine and co-developing training in clinical supervision and assessment.

Between 2017 and 2020, she coordinated a multi-country research programme on patient education and counselling for non-communicable diseases, supporting translation of evidence into policy and practice for hypertension and diabetes care in sub-Saharan Africa. She also coordinated postgraduate programmes within the Division of Family Medicine at Stellenbosch University.

In 2021, Dr Malan joined the University of Namibia, School of Medicine, as Head of the Division of Family Medicine. She led the development and implementation of Namibia’s Diploma in Family Medicine, served as Assistant Editor of the *African Journal of Primary Health Care & Family Medicine* and contributed to the Primafamed regional network. At the time of her passing, she was the country principal investigator for the National Institute for Health and Care Research (NIHR) funded Primary Care Assessment Tool (PCAT) project.

As a teacher and communicator, she embraced educational innovation. During the COVID-19 pandemic, she transitioned teaching to online platforms and introduced workplace-based assessment tools. Her impact was evident in tributes from students and colleagues.

Beyond her academic achievements, she will be remembered for her warmth, humour, individuality and collegial generosity. Dr Zelra Malan enriched countless lives and leaves an enduring legacy in African family medicine.

